# E-cigarettes may serve as a gateway to conventional cigarettes and other addictive drugs

**DOI:** 10.3389/adar.2023.11345

**Published:** 2023-06-30

**Authors:** Grace Chen, Shafiqur Rahman, Kabirullah Lutfy

**Affiliations:** ^1^ College of Osteopathic Medicine, Western University of Health Sciences, Pomona, CA, United States; ^2^ Department of Pharmaceutical Sciences, College of Pharmacy, South Dakota State University, Brookings, SD, United States; ^3^ College of Pharmacy, Western University of Health Sciences, Pomona, CA, United States

**Keywords:** adolescents, e-cig, smoking cessation, gateway, addictive drugs, conventional cigarettes, alcohol, cannabis

## Abstract

Electronic cigarettes (e-cigarettes) are devices that allow the user to inhale nicotine in a vapor, and are primarily marketed as a means of quitting smoking and a less harmful replacement for traditional cigarette smoking. However, further research is needed to determine if vaping nicotine via e-cigarettes can be effective. Conversely, nicotine has been considered a gateway drug to alcohol and other addictive drugs and e-cigarettes containing nicotine may have the same effects. Previous reports have shown that e-cigarette use may open the gate for the use of other drugs including conventional cigarettes, cannabis, opioids, etc. The increasing prevalence of e-cigarettes, particularly among youth and adolescents in the last decade have led to an increase in the dual use of e-cigarettes with alcohol, cannabis, and other illicit drug use like heroin and 3-4-methylenedioxymethamphetamine (MDMA). The advent of e-cigarettes as a device to self-administer addictive agents such as cocaine and synthetic cathinones may bring about additional adverse health effects associated with their concurrent use. This review aims to briefly describe e-cigarettes and their different generations, and their co-use with other addictive drugs as well as the use of the device as a tool to self-administer addictive drugs, such as cocaine, etc.

## Introduction

E-cigarettes were first introduced to the U.S. in 2006, primarily marketed as a means of smoking cessation and a less harmful replacement for traditional cigarette smoking [1]. In its U.S. patent (No. 8,490,628 B2), the e-cigarette is described as “An electronic atomization cigarette that functions as substitutes for quitting smoking and cigarette substitutes includes a shell” [2]. Since their introduction a decade and a half ago, e-cigarettes have undergone numerous structural changes, from early cigarette lookalikes called “cigalikes,” to modifiable tank e-cigarettes and, most recently, pod mods, better known as “pods,” which were first introduced in 2015 [3]. In 2018, JUUL, the most recognized pod, accounted for over three-quarters of the e-cigarette market [4]. The widespread popularity of e-cigarettes particularly among youth has accounted for an increase from 1.5% among youth in 2011 to 20.8% among youth in 2018 [5]. Several factors have increased the popularity of e-cigarettes among young adults in recent years [6–9], including their polished design, ease of use [10, 11], decreased aversive effects, wide variety of flavors [12], and the ability to be used discreetly [13–15]. The rapid rise of e-cigarettes preceded the research into the safety, efficacy, and effects on public health. Unlike conventional cigarettes, e-cigarette products evolve rapidly and vary in marketing practices between different countries, influencing how e-cigarettes are viewed and their impact in different areas [2]. This review aims to discuss the literature regarding the impact of e-cigarettes as a gateway to the use of conventional cigarettes, cannabis, alcohol, and other addictive drugs. The effect of e-cigarettes on cathinones, a new class of abused substances is also discussed.

## E-cigarette and conventional cigarette use

Along with the toxic effects of e-cigarettes, its pharmacological effects may also extend to its potential as a gateway drug for conventional cigarette use. While e-cigarettes have traditionally been hailed as a safer alternative to conventional cigarette use, recent studies have shown that e-cigarette users are 3–4 times as likely to begin using conventional cigarettes [16–18]. Additionally, many e-cigarette brands have been shown to contain more nicotine than claimed by the manufacturer, even among nicotine-free e-cigarettes; studies have shown that these brands may contain up to between 92% and 104% more nicotine than stated on the packaging [19, 20]. As nicotine has been shown to have multifactorial effects on brain development, such as those involved in mood disorders, attention, learning and memory, and impulsivity, this increase in e-cigarette use thus makes adolescents more prone to become users of conventional cigarettes, drugs, and alcohol [21]. Nicotine is the primary ingredient involved in the craving and withdrawal effects in both conventional cigarette and e-cigarettes. The drug acts primarily via activation of nicotinic acetylcholine receptors (nAChRs) in the brain and causes release of dopamine into the nucleus accumbens (NAc) [22–24]. Interestingly, while e-cigarettes were initially touted as a means of smoking cessation, they may actually be perpetuating increased nicotine use. A survey from the National Youth Tobacco Survey from 2011 to 2017 found that around 50% of adolescents who used one tobacco product also used multiple other tobacco products [25]. A 2017 systematic review by the National Academic Press found that there was significant evidence linking e-cigarettes to increased conventional cigarette use in both adolescents and young adults [26]. There have been clear links demonstrating e-cigarette and conventional cigarette use. For example, a review in 2019 on e-cigarette research found that adolescents who had previously used e-cigarettes were 3.6 times more likely to smoke conventional cigarettes than those who had not [27–29]. However, users of conventional cigarettes are likely to be in the same environment, demographic circle, and have similar behavioral characteristics to e-cigarette users [30]. These factors likely indicate that e-cigarette users who go on to use conventional cigarettes are already more susceptible to nicotine use. A recent study examining the relationship between these two substances used a model that produced less-biased and confounding effects [30]. They found that e-cigarette use was correlated with being twice as likely to use conventional cigarettes [30]. However, e-cigarette usage was not associated with current conventional cigarette smokers [30]. The authors further explain that the risk factors surrounding e-cigarette and conventional cigarette use are very similar—peer use, impulsivity, family education and history of smoking, internalizing symptoms, illicit substance use, sensation-seeking behavior—suggesting that use of conventional cigarettes is likely completely due to an underlying propensity for nicotine use [30]. Thus, while literature regarding the use of e-cigarettes as a gateway drug exists on both sides of the argument, more research is needed to definitively evaluate the use of e-cigarettes as a gateway to conventional cigarette use.

## E-cigarette and alcohol use

Studies on adolescents have demonstrated a clear order of substance use, specifically from legal substances such as alcohol and cigarettes to cannabis and then to illicit drugs, such as heroin or another opioid, methamphetamine, or cocaine [31]. As stated above, the dramatic rise of e-cigarette use among adolescents has been attributed to several factors, including its relatively easy accessibility, flavoring, and marketing, as well as lack of knowledge regarding their adverse effects [32, 33]. Although e-cigarettes were marketed as a means of conventional cigarette smoking cessation, studies among adults concerning the impact of e-cigarettes in this regard have largely been negative or not been definitive, with most analyses reporting a lack of substantial studies and wide confidence intervals [27, 34–37]. While alcohol and nicotine are implicated as gateway drugs in adolescents and young adults, using one before the other differs among ethnic and cultural demographics [38]. For example, Black youth were less likely than other races to begin smoking and also become habitual smokers, but this would reverse into adulthood [39]. Similarly, while there was a positive correlation among Whites concurrent alcohol and nicotine use, this finding was not observed in a study with Black participants [40]. Another study found that while among most European countries tobacco use was a predictor of future alcohol use, Dutch girls had a converse relationship [38]. However, alcohol and nicotine are more commonly co-abused than used separately; a study of high school students found that 55% only drank alcohol, while 88% used both substances simultaneously [41]. Although alcohol is the most frequently used substance among adolescents, tobacco use has been shown to predict alcohol use than its reverse counterpart [38, 42].

Alcohol affects multiple neurotransmitter systems in the brain, including dopamine, γ-amino butyric acid (GABA), glutamate, and serotonin [43]. In particular, the increase in accumbal dopamine is implicated in nicotine reinforcement and its motivational effects. Alcohol also stimulates the mesolimbic dopaminergic neurons in the ventral tegmental area, causing increased release of dopamine in the NAc [44, 45]. Activation of dopamine receptors in this area will increase the likelihood that a person repeats a particular behavior [46]. As such, the development of alcohol dependence may derive from its motivational properties which create the desire to consume alcohol as a result of its reinforcing action in the NAc [46].

In the brain, nicotine primarily serves to activate the nicotinic acetylcholine receptors (nAChRs) and stimulate dopamine release in the NAc, thereby increasing both cholinergic and dopaminergic neurotransmissions. It also causes the release of norepinephrine and serotonin, as well as affecting brain areas, such as the basal ganglia, hippocampus, and prefrontal cortex [24]. Alcohol affects a wide range of areas and neurotransmitters in the CNS, notably exciting the GABAergic system, modulating the dopaminergic system, inhibiting the glutamatergic system, and affecting the serotonergic system, as well as brain areas, such as prefrontal cortex, and limbic systems [47]. The large amount of systems affected by nicotine and alcohol play a role in not only in the effect of individual drug, but also the effect of the combined drugs and in potentiating the effect of each other. In rats, concurrent ingestion of nicotine and alcohol was shown to create an additive increase in the dopamine release in the NAc [48]. Nicotine was shown to cause the release of stress hormones such as glucocorticoids in the VTA to regulate the GABAergic activity induced by alcohol, thus reducing alcohol’s dopaminergic signals. This reduced dopamine signal has been associated to increased likelihood for alcohol and drug abuse as well as increased impulsivity; in other words, disruption of the GABAergic system has been positively associated with increased alcohol consumption [49, 50]. This demonstrates that the use of nicotine may reinforce the addictive effects of alcohol.

The impact of e-cigarettes as a gateway drug into alcohol use has been less studied, primarily due to the recent rise in adolescent e-cigarette use [51, 52]. Among adults, studies observing the impact of e-cigarettes on concurrent alcohol use have been varied, largely due to whether the increase in alcohol was due to conventional cigarettes or e-cigarettes. Studies have found that either nicotine product contributed to alcohol consumption [53], while others have demonstrated that e-cigarette users are more likely to consume higher levels of alcohol [54]. Conventional cigarettes were more likely than e-cigarettes to be concurrently used with alcohol in social settings [55], and heavy drinking was linked to individuals who consumed both e-cigarettes and conventional cigarettes, as compared to those who solely used e-cigarettes [56]. Finally, studies on adolescents who were heavy drinkers did not find a difference between those who used conventional cigarettes and those who used e-cigarettes [56]. Despite the variability regarding the influence of e-cigarettes on alcohol consumption, alcohol has been associated with increased pleasure when used in conjunction with nicotine. The range of data highlights a need to investigate further the relationship between e-cigarettes, alcohol, and conventional cigarettes.

Unhealthy drug use often occurs together in adolescents, i.e., cigarette use and alcohol consumption is commonly associated with behaviors such as unprotected sex, violent and criminal behavior, antisocial activity, and sedentary lifestyles [57, 58]. Alcohol, in particular, is a high health risk among adolescents; the Youth Risk Behavior Surveillance System 2017 survey reported that among high school students, 30% had consumed alcohol recently, 17% were in a car with someone who had consumed alcohol, 14% were binge-drinkers, and 6% had driven while drinking. Consequences from these behaviors manifest as brain developmental changes, failing in school, unprotected sex, legal issues, assault, and abuse of other drugs [59].

A meta-analysis comparing statistics among students who drank alcohol and concurrently used e-cigarettes concluded that efforts to curtail adolescent e-cigarette use should also simultaneously focus on stopping adolescent alcohol use [20]. Adolescent e-cigarette users were 6.5-times more likely to drink alcohol as well as meet the criteria of drunkenness and binge drinking [20]. This clustering of risky behaviors (e.g., alcohol and e-cigarettes) has been postulated to occur because they cover the same physical and psychological niche [60–62]. The gateway theory states that early use of cannabis, cigarettes, and alcohol progresses to more illicit substances in adulthood [63, 64]. Therefore, this pattern of use may lead to adolescent use of multiple substances to increase experimentation, risk-taking, and sensation-seeking [60, 65, 66]. Thus, the concurrent use of alcohol and e-cigarettes have combined pharmacological effects, which are postulated to activate the reward system and decrease withdrawal symptoms [67].

College-aged students are similarly susceptible to this dual risky behavior, with e-cigarette use increasing greatly in the past few years [68, 69]. As stated above, factors for the popularity of e-cigarettes among college students include more successful marketing, college-aged students being potential leaders in substance abuse patterns, and the novelty and flavors of e-cigarettes [68–70]. College students may be more susceptible to the synergistic effects of alcohol and cigarette use, which have traditionally been well-researched [71, 72]. E-cigarette use is more common among college students who already drink and use nicotine products [73, 74]. Among these studies, there is a positive association between binge drinking and e-cigarette use, although the motivations and risk perceptions were not analyzed. A meta-analysis examining the use of e-cigarettes and alcohol, as well as perceptions towards both, found that high alcohol consumption was positively correlated with a larger desire to try as well as continue smoking e-cigarettes [75]. College students who concurrently drank alcohol and used e-cigarettes cited similar reasons for using the latter, such that e-cigarettes were more acceptable, less toxic, and could be used for smoking cessation. These data parallel the research regarding the concurrent use of conventional cigarettes and alcohol among the general population [73]. The simultaneous use of alcohol and e-cigarettes may thus increase the risk among adolescents and young adults, a population vulnerable to risk-taking behaviors [76]. These data suggest that, unlike adult smokers, college-aged individuals do not use e-cigarettes as a means to quit. When examining e-cigarette use among drinkers, it appears that the motivation lies in the use of e-cigarettes as a method to receive nicotinic reinforcement while drinking in areas where conventional cigarette use is prohibited [70, 75]. This is supported by the fact that college students are more likely to endorse e-cigarettes as a more socially acceptable vehicle [69, 77]. Thus, e-cigarettes appear to cover a niche among concurrent alcohol and nicotine users that conventional cigarettes cannot provide.

## E-cigarettes and cannabis use among adolescents

In the United States and worldwide, cannabis is the most widely consumed illicit substance [78]. While delta-9-tetrahydrocannabinol (THC), the primary psychoactive ingredient in cannabis, has been demonstrated to increase dopamine release upon acute administration, its chronic use has been shown to blunt the rise in dopamine [79–81]. Initiation of use begins most often during the adolescent period, and research has demonstrated a link between heavy cannabis use and adverse health and social outcomes in adulthood [31, 82–86]. For example, cannabis has been shown to adversely affect neurocognitive function, causing learning difficulties, memory impairment, and lower attention and coordination rates [87, 88]. Higher levels of cannabis use during adolescence have been linked to higher rates of additional substance abuse, lower levels of wellbeing, increased risk-taking, higher levels of delinquency by age 20, and more difficulty in adulthood [89, 90]. Cannabis use has been linked to increased use of cocaine, prescription opioids, and alcohol [91–93]. Even occasional cannabis use has been linked to lower levels of educational attainment and a higher risk of escalation to the use of more illicit abused drugs [94]. Risk factors leading to cannabis use in adolescence include influence by peers, home environment, parental history and monitoring, difficulties in school, personality traits, disinhibition of behavior, as well as externalizing behavior [95–102].

Delta (9)-THC, the active ingredient of cannabis, interacts with G-proteins CB1 and CB2 [103]. CB1 mediates most of its psychoactive effects in the brain, and is found in the forebrain, midbrain, and hindbrain—areas associated with control of higher cognitive functions, motor control, and autonomic motor and sensory functions, respectively [103]. Namely, it is the interaction with the forebrain receptors which are thought to potentiate the reward circuits which are responsible for self-administration behavior, as well as the pleasurable and anxiolytic effects [104]. Cannabis has been shown to increase and alter dopaminergic activity in the VTA through the involvement of endogenous opioids [104]. It is believed that cannabinoids either act directly on the dopaminergic neurons or the neural circuits in the VTA [105, 106]. The use of cannabinoid agonists was also shown to reduce the release of GABA in the NAc, thereby disinhibiting dopaminergic neurons from the influence of GABAergic interneurons in the VTA and exert their rewarding effects [107–109].

While studies on the summative effects of concurrent nicotine and cannabis use are limited, studies have shown improved cognition in humans and rat studies [110]. This is hypothesized to be due to nAChRs and endocannabinoid receptors overlapping in the corticolimbic regions of the brain, such that activation may serve to dampen some of the effects when these substances are used alone [88, 111, 112]. Additionally, it is hypothesized that cannabis and nicotine may have a synergistic effect on the dopaminergic inputs to the limbic and cortical areas [113, 114]. However, studies on the effects of concurrent nicotine and cannabis use are preliminary, and it is hypothesized that continued co-use is more likely to lead to adverse outcomes [115, 116].

Rates of cannabis use have been rising among adolescents; it is estimated that up to one-third of this demographic will have tried marijuana by the time they graduate [117]. In the U.S., increases in the legality of cannabis have made the drug more accessible to individuals. Adolescents have been shown to view cannabis in a more positive light and have less knowledge about its risk factors [78, 118]. As a result of this increased accessibility, almost twenty percent of adolescents between 13 and 17 admit to frequent use of cannabis [90]. In the face of the adverse effects precipitated by early use of cannabis, the addition of e-cigarettes poses a possibly additive risk for these young individuals [119].

E-cigarettes and cannabis have seen similar trends in use among adolescents. As these two substances are the most commonly used drugs during adolescence, their co-use has provided ways of examining the adverse effects of the co-use. An e-cigarette user is also highly likely to be a cannabis user [120, 121]. A national study among high school students found that almost 40% of current users of e-cigarettes also used THC as an ingredient in their e-cigarettes [122]. In another study, up to 27% of high school e-cigarette users also were using the device to vape cannabis [123]. With the rise in use of e-cigarettes, manufactures are producing products that allow consumption of both products, such as pen-sized vaporizers which are specially designed for dual use. As cannabis is usually vaporized at lower temperatures than e-cigarettes, the higher temperatures reached in these products also propose possible health consequences [106]. Additionally, as these products are unregulated and currently under-researched, the lack of quality control proposes the possibility of adverse effects in users [124]. Studies have shown that compared to nonusers, e-cigarette users were three times more likely to use cannabis [125]. In another study, the use of conventional cigarettes, e-cigarettes, or hookah—all nicotine-containing devices—was linked to the concurrent use of cannabis after 2 years [126]. The increase in vaping as means to consume cannabis has risen as well, with 12.4% of 10th graders and 13.1% of 12th graders vaping cannabis in the past year [119]. The trajectory of e-cigarette use and vaping cannabis have similar patterns between the 11th and 12th grades, with both demonstrating increasing use as well as their co-use being more popular among heavier users [127]. While research on the health effects regarding the concurrent use of cannabis and e-cigarettes is still largely unknown, emerging studies have shown that the concurrent use of these products leads to increased substance use and mental health problems, learning and memory problems, nicotine addiction, difficulties in tobacco smoking cessation, as well as lower motivation for cessation [128, 129]. In addition to these internal problems, the concurrent use of cannabis and e-cigarettes has also been linked to an increased risk of unintentional injuries and risky behavior, such as driving while intoxicated, binge drinking, and abuse of prescription drugs and alcohol [119].

The combinative use of electronic nicotine delivery systems (ENDS) and cannabis also pose many health and social adverse effects. Among ENDS users, cannabis use was related to increased e-cigarette use, anxiety, and problems related to ENDS, while among cannabis users, ENDS use was linked to higher levels of depression, anxiety, and problems related to cannabis [130]. As of February 2020, there have been a total of 2807 cases and 68 deaths linked to e-cigarette or vaping use-associated lung injury (EVALI), which is a clinical diagnosis necessitating e-cigarette use within 90 days before pulmonary symptoms, infiltrates on chest X-ray or CT not explained by secondary factors [131]. While initially believed to be a result of e-cigarette vaping, these deaths were later attributed to the additive vitamin E-acetate, found in unregulated cannabinoid fluids [132, 133]. Dual users of ENDS and cannabis may be exposed to higher levels of carbon monoxide, although neither seem to increase the metabolism of the other. In animal studies conducted with smoke machines, tobacco smoke alone contained higher amounts of its common ingredients, such as nitrosamines and formaldehyde versus cannabis, which contained higher quantities of other substances, such as ammonia and tar. While not studied in combination, these results may indicate that dual use facilitates exposure to higher amounts of toxins [134]. There is currently still a dearth in research regarding the health effects of additive marijuana and nicotine use, but the literature indicates that use of one often implicates the use of another. Additionally, with the legalization of cannabis in 2018, smoke shops have began to carry e-cigarette devices meant to also vape cannabis oil and marijuana [135]. THC derivatives, such as delta-8-THC and delta-10-THC, have been mixed into products marketed as cannabis. These isomers are found in smaller amounts in cannabis, and importantly, are currently federally unregulated. The current research on delta-8-THC and delta-10-THC is minimal [136], and the FDC reports that there were 104 reports of adverse events in users of delta-8-THC. These unregulated substances, which are becoming widely prevalent, may have further consequences on patient safety [137–139].

In contrast, individuals who used cannabis or e-cigarettes exclusively demonstrated a lower risk of participating in such behaviors, thus highlighting the potentially additive adverse effects of the co-use [119]. Concurrent users of cannabis and e-cigarettes were also more likely to engage in risky sexual behavior, as dual users were more likely to be sexually active and have higher rates of lifetime sexual partners [119]. These data corroborate the published data detailing the adverse effects of tobacco and cannabis use, such as worsening mental health and decreased neurocognitive function [115]. Thus, the increasing popularity and availability of e-cigarettes and cannabis, combined with users’ shared risk factors, may be contributing the concurrent usage of both substances.

## E-cigarettes and illicit drug use

Illicit drugs are defined as drugs that are used for non-medical purposes due to the high risk of abuse [140]. For over half a century, plant-based drugs such as cannabis, cocaine, and synthetic drugs like heroin, α-pyrrolidinopentiophenone (α-PVP), 3,4-methylenedioxypyrovalerone (MDPV), 3,4-methylenedioxy-metaphetamine (MDMA) and amphetamines, and medications like benzodiazepines, methadone, and buprenorphine have been under strict international control [141]. The rise of e-cigarettes have preceded an incidence in the concurrent use of such drugs through the means of inhalation.

In the past decade, there has been more than a ten-fold rise in the transition from conventional smoking to vaping in the United States [142]. As of 2014, e-cigarettes became more popular than traditional cigarettes among US youths [143]. Similarly, 37% of students ages 15–16 in Wales, United Kingdom, had ever used e-cigarettes versus 26% of students who used conventional cigarettes [144]. The drug delivery system of e-cigarettes has experienced a change throughout the generations since the first-generation e-cigarette, which conventionally had the appearance of a cigarette [145]. Second-generation e-cigarettes introduced refillable fluid tanks, a change in style, and bigger rechargeable batteries [146]. Third-generation e-cigarettes made it possible to change the voltage to modify the atomizer temperature, changing the ability to modify how much vapor could be produced [124, 146]. Additionally, these e-cigarettes came equipped with a larger size and battery capacity, which allowed for increased liquid storage of e-cigarette vapor [146]. Fourth-generation e-cigarettes contain nicotine salts, including disposable e-cigarettes such as Juuls, and allow for higher heating coil temperatures [147]. These changes reflect the increasing customizability allowed via this inhalation method.

With the increasing popularity of e-cigarettes comes a consequential increase in the alternative uses of this technology and the question of whether other illicit drugs may be consumed via a similar method [148, 149]. Cannabis vaporization has existed for a long time, and studies have shown that medical cannabis vaporization can produce similar blood concentrations of THC as conventional cannabis smoking [150–152]. An examination of the pharmacokinetics and pharmacodynamics of other drugs of abuse indicates that the delivery system provided by e-cigarettes allows for a similar route of administration [153]. By definition, “vaping” describes inhalation of a substance through the mouth using a device that is electrically powered to such that it is vaporized and consumed. Examples of well-known products include nicotine dissolved in e-liquids using a mixture of glycerine and propylene glycol as well as concentrated plant material extract [153]. While the literature on the mechanism-of-action of cannabinoid and nicotine is well established, there lies much potential and harm in the ingestion of alternative illicit drugs via this means of delivery.

## 3-4-Methylenedioxymethamphetamine (MDMA)

Colloquially known as “molly” and “ecstasy,” 3-4-Methylenedioxymethamphetamine (MDMA) is a central nervous system (CNS) psychostimulant and derivative of amphetamine that is commonly used to increase feelings of euphoria and empathy [154]. It works by blocking the reuptake of monoamine neurotransmitters such as dopamine, norepinephrine, and serotonin—particularly the latter two. Additionally, MDMA further decreases monoamines’ reuptake by reversing monoamine transporters and serves as a competitive substrate [155–158]. Frequent usage of MDMA leads to rapid tolerance and increased adverse events; however, these toxic effects and dosages are customized based on individual physiology and susceptibility [159]. Regarding its use as an inhaled device, up to 11.7% of individuals who vaped have also vaped MDMA [160]. On internet forums, users have described the use of vaporization techniques, including tabletop vaporizers and e-cigarettes, to deliver the drugs. Of note was that some users made sure to convert MDMA into a freebase form before inhalation [153]. While there is still no definitive literature regarding the concurrent use of e-cigarettes and MDMA, a similar route of administration and neurochemical interactions in the brain may contribute to a dual use.

## Synthetic cathinones

Synthetic cathinones are a growing new family of psychoactive substances, and are seen as an alternative to amphetamines and cocaine [161]. Up to 30% of new psychoactive products were labelled as synthetic cathinones, and are commonly known as “bath salts,” “research chemicals”, and “plant foods” [161–163]. The lack of quality control in its manufacturing makes it difficult to determine chemical purity, with the majority of these substances having more than one psychoactive ingredient [164, 165]. In the US, popular synthetic cathinones include pentedrone, MDPV, and α-PVP. Similarly to MDMA and amphetamines, they work by inhibiting norepinephrine, serotonin, and dopamine transporters [163]. Interestingly, because these drugs vary in their affinity for these monoamine transporters, their mechanism of action and their effect vary across different products [166]. Additionally, these products also function as releasers of monoamines, with likely different effects on different neurotransmitters [163]. These drugs are associated with rapid onset of action depending on method of administration, ranging from minutes to hours [163]. Their reported effects are similar to those of amphetamines, and include increased empathy, focus, sociability, sexual arousal, and euphoria [163, 164]. The method of smoking or vaporizing these synthetic cathinones was first published in 2012–2013, around the time when e-cigarette devices were becoming popular [130]. It was demonstrated that vaporizing these products allowed for a faster onset of action, shorter duration, and quick onset of effects compared to the more common “snorting,” or nasal inhalation [112]. Thus, e-cigarettes are being used as a means to vaporize such drugs with positive feedback from users [55]. However, similarly to cannabis, the heating of these commonly “snorted” stimulants may produce different psychoactive and toxic metabolites as a result of thermolysis [112]. Earlier studies examining the thermolytic products of methamphetamine and synthetic cathinones have been linked to substances which cause respiratory irritation, tachycardia, hypotension, and bronchoconstriction [167–169].

α-PVP is more potent than amphetamines or cocaine at the dopamine and norepinephrine transporters, and this drug has been linked to at least 23 deaths between 2011 and 2015 [161, 164, 170]. These drugs are administered via different routes, including oral, injection, snorting, smoking, inhaling, rectal, and sublingual [170]. Research on drug forums has shown that the use of e-cigarettes to vaporize these products has been linked to faster onset as well as a more rapid duration of action [160]. Up to 7.1% of e-cigarette users have been reported to have vaped α-PVP [171].

Mephedrone inhibits serotonin, norepinephrine, and dopamine transporters [164, 172], and has a rapid duration of action, allowing for 1 g–2 g to be consumed in a single episode with effects lasting up to 4 h [164, 173]. At least 12 fatalities related to mephedrone have been reported [173]. Side effects include fluctuating body temperatures, mydriasis, blurry vision, agitation, hypertension, chest pain, paranoia, suicidal ideation, paranoia, and psychosis [161, 173, 174]. Up to 8.5% of e-cigarette users have reported vaping mephedrone [171].

While MDPV does not influence neurotransmitter release, it is an inhibitor at dopamine and norepinephrine transporters [161, 162, 166, 170, 175]. Side effects of MDPV use include acute kidney failure, psychosis, paranoia, rhabdomyolysis, metabolic acidosis, and hyperthermia [176]. Reports also exist showing that MDPV is consumed via vaporization [177] Thus, while definitive literature regarding the dual use of e-cigarettes and synthetic cathinones α-PVP, mephedrone, and MDPV is still premature, similarities in their mechanism of action and route of administration may play a role in their current use.

## Cocaine

Cocaine, commonly known as “coke” in its salt form and “crack” in its free base form, is a CNS and peripheral nervous system (PNS) stimulant, is the second-most abused illicit drug in the world [178]. Its mechanism of action is via blockage of the dopamine, norepinephrine, and serotonin transporter [178, 179]. In the limbic system, the blockade of DA transporter in the NAc has been associated with the feelings of pleasure generated by cocaine ingestion [180]. In addition, the drug also affects the electrical conduction of the heart, blocking voltage-gated sodium channels [181]. Users report that “crack” is a more impure version of cocaine, thus distinguishing it from its counterpart free base cocaine [153]. Cocaine is administered through inhalation and intravenous methods and has a rapid duration of action, thus leading to higher levels of user redosing [182, 183]. This leads to a greater risk of drug dependence and toxicity [182]. Additionally, the toxic effects of cocaine are related to user susceptibility, tolerance, and route of administration, with intake of more than 1 g demonstrating fatality [184]. Side effects of cocaine usage include ventricular fibrillation and tachycardia, seizures, myocardial infarction, cerebrovascular accident, violent behavior, QRS prolongation, delirium, respiratory arrest, anxiety, and muscle rigidity [185]. When compared with hydrochloride salt of cocaine, the use of “crack” is associated with increased aggression and violent behavior [182].

In a 2012 survey of US adults aged 18 to 34, about 88% of those who used cocaine at least once had smoked cigarettes previously, 5.7% began both substances simultaneously, 3.5% started with cocaine first, while 2.9% had never smoked cigarettes before using cocaine [186]. In a study on dogs, the combined use of cocaine and nicotine produced synergistic effects such as increased heart rate and blood pressure when nicotine was administrated after cocaine; however, the excitatory effects produced by nicotine was decreased by cocaine use [180]. Similarly, a study on rats demonstrated that pretreatment with a nicotine patch decreased rats’ “high” and “stimulated” behavior, and increased the time to detect the euphoric effects of cocaine; conversely, nicotine did not have an effect on the physiological effects of cocaine [187]. The pretreatment of nicotine or cocaine before the other has shown to have varying effects [188]. Preclinical studies have shown that animals pretreated with nicotine in early adolescence demonstrate increased rewarding effects of cocaine as well as locomotor sensitization [189–192]. In another study, pretreatment with nicotine reduced the rewarding effect of cocaine in adult mice [193]. Thus, while there is research demonstrating a positive synergistic process between these substances’ dual use, more research is required to better observe the health effects on humans.

A positive association has long been established regarding cocaine and conventional cigarette use. In a study from 1990, data showed that compared to individuals who did not use drugs, those who used cocaine were at higher likelihood to smoke cigarettes [194]–up to 3–4 times more likely [195, 196]. Conversely, the use of stimulants has been associated with increased use of conventional cigarettes and nicotine [197, 198]. In animal studies on monkeys and Sprague-Dawley rats, cocaine has been shown to serve as a substitute for nicotine, as well as the reverse, although results have produced variable outcomes [199–203]. Preclinical studies on rats showed the prior nicotine exposure increase cocaine self-administration but this was only observed in adolescent but not adult rats [204]. However, pretreatment with nicotine in adult mice showed decreased cocaine-induced condition place preference, and the robustness of the response was dependent on nicotine dosage [193]. Clinical research regarding the link between conventional cigarette and cocaine use has similarly produced mixed results. A 1996 study investigating cocaine-dependent smokers and non-smokers found that the former reported spending more money and using more cocaine per week [205]. Studies comparing subjects’ physiologic and subjective responses found that nicotine enhances cravings for cocaine [206], while another found similar responses to both substances [207]. A questionnaire focused on asking users whether nicotine enhances desire for cocaine and *vice versa* concluded that the cigarette smoking may positively influence the high and cravings caused by cocaine use [208]. The concurrent usage regarding cigarette smoking and cocaine use may be due to the neurochemical similarities in that both substances alter the brain’s dopaminergic activity, specifically increasing dopaminergic release in the NAc [209–214]. These similarities thus may be carried over to the concurrent usage of e-cigarettes and cocaine.

A prospective cohort study in the United Kingdom followed over five thousand youth regarding the association between e-cigarette and subsequent cocaine use. They found that youth who had used e-cigarettes before 14 years of age were 2 times more likely to use cocaine (7.6% versus 3.1%) when matched with non e-cigarette users [215]. While definitive literature implicating e-cigarettes as a gateway drug for cocaine use is still unestablished, studies on adolescent rats found that pretreatment with nicotine increased self-administration of cocaine and also increased cocaine reward [204, 216]. The connection between cocaine and e-cigarettes lies in the usage of “crack” cocaine, which is able to be purchased on the “dark web,” a subset of the internet that cannot be easily accessed by governmental agencies [217]. In user surveys, up to 8.4% and 10.9% of e-cigarettes users had vaped crack cocaine and cocaine powder, respectively [160]. Of these, 74% preferred to use e-cigarettes as their means of administration [160]. Cocaine decomposes at the required vaporization temperature required of e-cigarettes; however, hydrochloride salt of cocaine has a melting point of 195°C [218]. However, the free base form of cocaine only decomposes at 200°C, and cocaine can be volatilized to this free base form from the hydrochloride salt form at 100°C [149]. The use of thermolytic degradants with cocaine and methamphetamine products, however, has been associated with carcinogenic elements and psychoactive pyrolysis substances [171]. Thus, while the literature between e-cigarette and cocaine use is still limited, a well-documented positive association with conventional cigarettes as well as similar neurochemical properties may provide future revelations on their concurrent use and influence.

## Opioids

### Heroin

Heroin is a Schedule I drug associated with up to 90,000 total US deaths following its intravenous, snorting, and inhalation route of administration [159, 219, 220]. In 2020 the CDC reported that over 13,000 individuals died using drugs containing heroin, which amounts to over 4 per 100,000 individuals [233]. This reflects a greater than 7-fold increase between 1999 and 2020 [233]. After crossing the blood-brain-barrier, heroin is converted into 6-monoacetylmorphine (6-MAM) and subsequently morphine [220,221]. Additionally, it is also converted into other substrates such as morphine-3-glucuronide (M3G), a toxic substrate [220]. Morphine, 6-MAM, and heroin all have an affinity for the mu-opioid receptor, and have similar effects [221]. Side effects of heroin include leukoencephalopathy, coma, pulmonary edema, seizures, sinus tachycardia, paranoia, agitation, hallucinations, and sudden death [221]. The term “chasing the dragon” is century-long known method for its ingestion, in which users use a heated metal surface, such as a spoon, to inhale its vapor [222]. However, the side effects and toxicities are dependent on user susceptibility and duration of usage. Vaporizing heroin at higher temperatures produces pyrolysis substances and side effects including encephalopathy and acute eosinophilic pneumonia [149, 223]. Analysis of drug forums has shown heroin in its free base form is also ingested via e-cigarette devices, and that more than 7% of electronic vaping users had ingested heroin through these means [160].

### Fentanyl

Fentanyl an opioid receptor agonist used as a preanesthetic agent is involved in more than 70% of all opioid-related deaths [224,225]. In 2021, the US reported 71,238 deaths due to opioids, which is up from 57,834 in 2020 [234]. Up to 100 times more potent than morphine, fentanyl diffuses quickly through the body’s membranes. As a lipophilic substance, fentanyl’s pharmacokinetics vary largely in individuals depending on their level of adipose tissue [226]. The effects of fentanyl are similar to those of opioids used to induce drowsiness, euphoria, anxiolysis, and analgesic effects [227]. Side effects of fentanyl usage include confusion, pruritus, nausea, orthostatic hypotension, constipation, seizures, weakness and hallucination [228]. Causes of fentanyl overdose include respiratory arrest, extreme fatigue and confusion, obtundation, bradypnea, and cardiac arrest [229]. In the context of inhalation of fentanyl, 7.3% of e-cigarette device users reported vaping fentanyl [160]. Case reports including inhaled fentanyl intoxication of its derivative 4-fluorobutyrfentanyl (4-FBF) as well as a young adult with 4-FBF and an e-cigarette near his body both showed blood compositions of e-cigarette fluid and 4-FBF [230]. Another case report describes a 36-year old male with the primary complaint of altered mental status reporting usage of acetylfentanyl [231].

Thus, while the use of these opioids with e-cigarettes requires more studies, these documented instances of concurrent heroin and fentanyl usage with e-cigarettes provides an opening to possibilities regarding their dual usage.

## Concluding remarks

This review aimed at exploring the effect of e-cigarettes on the concurrent use of other drugs of abuse, particularly among adolescents and young adults, as well as provide insight into shared characteristics predisposing individuals to their concurrent usage. E-cigarettes have emerged onto the market as an easily accessible and attractive means of consuming nicotine. With few governmental regulations regarding their distribution, marketing, and legality, e-cigarettes have become commonly used among adolescents and young adults. The adverse effects of nicotine on the developing young adult brain have been well-studied, as well as the effect on the brain dopaminergic pathway produced by conventional cigarettes and e-cigarettes. As nicotine has been implicated as a gateway drug into other addictive drugs, the rise of e-cigarettes has also given way to the concurrent usage of other substances, such as alcohol and cannabis. While literature on whether e-cigarettes actually predispose individuals to other drugs is still variable, there have been documented concurrent usage with these drugs, possibly due to a similarity in risk-factors and neuronal pathways. The properties of nicotine are well-documented for over a century, including its adverse effects on the brain and the consequences on adolescent and young adult use. E-cigarettes have been shown to serve as a vehicle for the inhalation of other drugs, such as MDMA, synthetic cathinones, cocaine, and opioids, which may further encourage the concurrent usage of these drugs. One potential target for the gateway effect of conventional cigarettes and potentially that of e-cigarettes is the rise in dopamine in the NAc, as most drugs abused by humans increase the level of dopamine in this brain area [211] and has been implicated in their pleasurable effects and in the initiation and maintenance of substance use disorders. Indeed, an earlier microdialysis study showed that co-administration of nicotine and alcohol increases accumbal dopamine to a greater level than each drug alone, a synergistic effect [232]. Thus, while e-cigarettes are marketed as an alternative strategy for curbing nicotine addiction, the rise of e-cigarette use in adolescents and younger adults makes it a serious contender as a gateway towards other drugs of abuse.

## Abbreviations

E-Cigarettes; e-cigarettes, Electronic cigarettes; nAChRs, nicotinic acetylcholine receptors; NAc, nucleus accumbens; GABA, γ-amino butyric acid; THC, tetrahydrocannabinol; α-PVP, α-pyrrolidinopentiophenone; MDPV, 3,4-methylenedioxypyrovalerone; MDMA, 3,4-methylenedioxy-metaphetamine; CNS, central nervous system; PNS, peripheral nervous system; 6-MAM, 6-monoacetylmorphine; 4-FBF, 4-fluorobutyrfentanyl.
